# Quantifying task-related gaze

**DOI:** 10.3758/s13414-024-02883-w

**Published:** 2024-04-09

**Authors:** Kerri Walter, Michelle Freeman, Peter Bex

**Affiliations:** Department of Psychology, Northeastern University, Boston, MA USA

**Keywords:** Visual search, Eye movements: cognitive, Natural image statistics

## Abstract

Competing theories attempt to explain what guides eye movements when exploring natural scenes: bottom-up image salience and top-down semantic salience. In one study, we apply language-based analyses to quantify the well-known observation that task influences gaze in natural scenes. Subjects viewed ten scenes as if they were performing one of two tasks. We found that the semantic similarity between the task and the labels of objects in the scenes captured the task-dependence of gaze (t(39) = 13.083; p < 0.001). In another study, we examined whether image salience or semantic salience better predicts gaze during a search task, and if viewing strategies are affected by searching for targets of high or low semantic relevance to the scene. Subjects searched 100 scenes for a high- or low-relevance object. We found that image salience becomes a worse predictor of gaze across successive fixations, while semantic salience remains a consistent predictor (X^2^(1, N=40) = 75.148, p < .001). Furthermore, we found that semantic salience decreased as object relevance decreased (t(39) = 2.304; p = .027). These results suggest that semantic salience is a useful predictor of gaze during task-related scene viewing, and that even in target-absent trials, gaze is modulated by the relevance of a search target to the scene in which it might be located.

## Introduction

### Factors influencing gaze in scenes

There are multiple factors that drive where we decide to focus our gaze in everyday scenes. Because of this, the study of eye movements has become a widely utilized method in a variety of research areas. Gaze control in static scenes is comprised mainly of two components, fixations (periods of time where our eyes are relatively still) and saccades (periods of time where eyes are moving ballistically to a new fixation location). The human eye obtains the highest resolution information at the center of gaze (the fovea). Because visual sensitivity is reduced during saccades (Dorr & Bex, 2013; Martin, 1974), the majority of spatial information is gathered during fixations. To obtain as much high-resolution information as possible, we move our eyes two to four times per second to fixate new locations in a scene (Buswell, 1936).

Eye movements are strongly linked to attention, as overt attention requires us to direct our gaze to an object in order to attend to it. Objects attract attention in scenes (Stoll et al., 2015) even without conscious effort (Kimchi et al., 2007), and further research suggests that this is exacerbated with unusual or incongruent objects (Henderson et al., 2019; Öhlschläger & Võ, 2017). Once fixated, attention spreads across the entire surface of the object (Duncan, 1984; Egly et al., 1994). Similarly, research shows that the viewer’s task also influences their eye movements (Castelhano et al., 2009; Hayhoe et al., 2003; Nuthmann, 2017; Rothkopf et al., 2016; Yarbus, 1967), as information in different discrete scene areas is necessary to complete different tasks (for a comprehensive review on visual search, see Eckstein, 2011). With these factors in mind, two prominent theories have been developed to explain what image features guide eye movements when viewing natural scenes: low-level image salience and high-level semantic salience.

### Image salience

Image salience theories posit that eye movements are guided by low-level, bottom-up factors such as variations in color, contrast, edge orientation, and brightness. Because evidence suggests that both overt attention (Parkhurst et al., 2002) and gaze (Borji et al., 2013; Harel et al., 2007; Itti & Koch, 2001; Parkhurst et al., 2002) are guided by areas of high visual salience (areas that have high local variation in the above mentioned image features), many researchers have developed models that successfully predict human gaze based on these low-level image salience features (Borji et al., 2013; Harel et al., 2007; Itti & Koch, 2001; Parkhurst et al., 2002; for review, see Pedziwiatr et al., 2021; Yan et al., 2021). For our experiments, we have elected to utilize graph-based visual saliency (GBVS; Harel et al., 2007), due to its availability as an open-source toolbox and its robustness and evaluated success in predicting human fixations. GBVS combines color, edge-orientation, and intensity conspicuity maps, to create salience heatmaps that predict human gaze.

### Semantic salience

Semantic salience theories argue that eye movements are instead guided by high-level, top-down factors. These factors rely more on higher level cognitive processing and require prior knowledge of the world. Evidence suggests gaze is guided by a setting’s context (Castelhano et al., 2009; Castelhano & Henderson, 2007; Hayes & Henderson, 2021; Henderson et al., 2019; Yarbus, 1967) and locations necessary for future action (Hayhoe et al., 2003; Johansson et al., 2001; Land et al., 1999). Additionally, research shows that semantically related search targets are easier to find in scenes (Biederman et al., 1982; Davenport & Potter, 2004; Henderson et al., 1999; Palmer, 1975; Rémy et al., 2014). Evidence also demonstrates that the general contextual concept of a scene, or “gist,” can be extracted upon first glance (Friedman, 1979; Oliva, 2005), and that this provides the viewer with predicted objects and their approximate spatial arrangements in a scene based on the prior knowledge of that type of scene (Henderson, 2003; Larson & Loschky, 2009; Nakamura, 1994). This prior knowledge has been shown to guide eye movements in various ways, such as directing gaze toward areas where a target object is likely to be found (Võ et al., 2019) and capturing the attention of objects that are inconsistent with their scene context (Bonitz & Gordon, 2008; Cornelissen & Võ, 2017; Henderson et al., 1999; Loftus & Mackworth, 1978; Võ & Henderson, 2009). Numerous researchers have developed models that successfully predict human gaze based on these high-level semantic salience factors (Henderson et al., 2019; Hwang et al., 2011; Nyström & Holmqvist, 2008; Onat et al., 2014; Rider et al., 2018; Rose & Bex, 2020; Stoll et al., 2015).

### Present study

In this study, we attempt to provide further evidence that task influences gaze, coupled with a semantic salience-based analysis to demonstrate that the semantic understanding required for task completion can be quantified with language models. We also adapted a standard present/absent visual search task using high and low semantic relevance targets to examine how image salience and semantic salience-based predictions of gaze vary during search. Following classic studies in the literature (e.g., Yarbus, 1967), we hypothesize that task will influence gaze, and furthermore, that gaze guidance is quantifiable with semantic context when performing a task. For our search task, we hypothesize that while image salience can be a reliable predictor of gaze guidance, especially at the start of a trial, its usefulness will decline once semantic understanding of the scene context is processed. Similarly, we hypothesize that search for high-relevance semantic targets will be better predicted by semantic-salience, while fixations for low-relevance scene targets will be better predicted by image-salience.

## Methods

### Apparatus

MATLAB (The MathWorks, Inc., Natick, MA, USA) with Psychtoolbox (Brainard, 1997) was used to program the experiment, and MATLAB’s Text Analytics and Statistics and Machine Learning toolboxes were used to perform the analyses and create figures. R studio was used for all linear model analyses. The experiment was run on a Dell OptiPlex 9020 desktop computer (Dell Inc. Round Rock, TX, USA) with a Quadro K420 graphics card (nVidia, Santa Clara, CA, USA). Stimuli were presented on a 60 cm x 34 cm BenQ XL2720Z LCD monitor (BenQ Corporation, Taipei, Taiwan) set to a screen resolution of 1,920 × 1,080 pixels at 120 Hz. A chinrest was utilized to stabilize the head position of participants, who were seated 63 cm from the screen (width = 50.9°). Eye movements were recorded using an Eyelink 1000 (SR Research Ltd. Mississauga, Ontario, Canada) with a sampling rate set to 1,000 Hz and utilizing the MATLAB Eyelink Toolbox (Cornelissen et al., 2002). We used the built-in Eyelink nine-point calibration and validation procedures at the beginning of the experiment and in between blocks. The eye-tracker error specified by the manufacturer was 0.375°, and this value was used to specify the standard deviation of Gaussian error for fixation processing.

### Stimuli

We used the LabelMe database (Russell et al., 2008) for selection of ten images (five indoor, five outdoor) for Experiment 1 and 100 images (50 indoor, 50 outdoor) for Experiment 2.The LabelMe database is a collection of natural scenes that have been manually annotated by human volunteers. Because the database is open-source, some noise arises from the use of human volunteers, mainly in the form of unnecessary labels or incorrectly labeled objects (Rose & Bex, 2020). To counteract this, we manually reviewed and revised our images to ensure accurate labeling (for details, see Walter & Bex, 2022). We only selected large, clear images that had at least 15 unique objects and 75% of the image surface labeled. Source images ranged from 1,365 x 1,024 pixels to 3,872 x 2,592 pixels and were resized to approximately 1,280 x 960 pixels onscreen with preserved aspect ratio and were presented in random order.

#### Image salience

To create the image salience maps, we ran our images through GBVS using the standard settings. GBVS creates three individual conspicuity maps: color, orientation, and intensity, and combines them to create a final heatmap of salient image features. Areas on the heatmap range from 0 (low image salience) to 1 (high image salience). Areas with high image salience are areas that differ strongly from their surroundings in the corresponding feature (e.g., color: bright red traffic light against green trees; orientation: sharp roof edge against plain sky; intensity: bright window in a dark room).

#### Semantic salience

To quantify semantic information, we utilize Global Vectors for Word Representations (GloVe; Pennington et al., 2014), a pretrained regression model, due to its extensive database and established utility in the literature. Semantic similarity is quantified by how near two words fall in semantic space, which GloVe creates by categorizing words along feature dimensions and connecting them as a similarity web. The GloVe model can then compute a similarity score (0 = not similar, to 1 = identical) by comparing the angles and vector lengths between words in this semantic space. If any object was comprised of two words (e.g., “window blinds”), the vectors for each word part were taken individually and combined to find the nearest common word, using Glove’s vec2word function (in this example, the nearest common word was “window”).

GloVe has been operationalized for visual search in the Linguistic Analysis of Semantic Salience (LASS) model (Rose & Bex, 2020) and is one of the more recent and frequently updated models to date. For this study, we used the pretrained word vector Common Crawl (840B tokens, 2.2M vocab, cased, 300d vectors), which can be obtained at https://nlp.stanford.edu/projects/glove/. To utilize GloVe with the LabelMe images, we used it in conjunction with LASS. LASS is computed in three steps: generating scene context labels (respective to task; see *Procedure* for details), generating scene object labels and masks (LabelMe), and calculating the semantic similarity scores between the object labels and context labels (GloVe) to then embed in the object masks, which assigns a similarity value to each pixel in the image for each scene label. The resulting product is a heatmap of semantic salience features where areas range from 0 (low semantic salience) to 1 (high semantic salience). Areas with high semantic salience are areas that are conceptually relevant in the scene, (e.g., a refrigerator in a kitchen; a car on a street; a bicycle in a park).

#### ROC analyses

In both experiments we utilized a receiver operating characteristic (ROC)-based analysis to determine the prediction power of our image salience or semantic salience heatmaps. ROC curves are created by assessing the number of true positives (hits), true negatives (correct rejections), false negatives (misses), and false positives (false alarms) at increasing salience levels. Each metric is classified as follows: true positives are areas of heatmap that the model predicted and were fixated, misses are areas of heatmap the model predicted and were not fixated, correct rejections are areas of heatmap that were not predicted and were not fixated, and false positives are areas of heatmap that were not predicted and were fixated. We used a Matlab AUC extension from Judd et al. (2012) called AUC_Judd, which calculates the true positive and false positive rate from the above-mentioned metrics. The true positive rate, or sensitivity, is equal to true positives / (true positives + false negatives). The false positive rate is equal to 1 – specificity, where specificity is true negatives / (true negatives + false positives). In this way, an ROC curve is generated from finding these values at increasing salience levels, and the area under the resulting ROC curve (AUROC) is the prediction power of the salience map, given a subject’s gaze.

### Participants

Forty subjects (31 female, nine male) between the ages of 17 and 20 years were recruited from the Northeastern University undergraduate population. Thirty-eight subjects received course credit as compensation for their participation, and two subjects received monetary compensation. Sample size was determined using a power analysis with the means of image salience and semantic salience and average standard deviation from previous work (Walter & Bex, 2022), an alpha of .05, and a desired power of .80. In order to maintain a balanced design, the total subject count needed to be a multiple of 4. Because the effect size of our previous results was high (d = 7.424), we decided to use a similar number of subjects (N = 30), but as a multiple of 4. Subjects were required to complete a demographic questionnaire and sign a consent form approved by the Institutional Review Board at Northeastern University before participation in this experiment. Thirty-one subjects reported English as their first language, and of the remaining nine, eight reported being fluent in English while one reported they were still learning English. However, none of the subjects reported difficulty in completing the task due to a lack of understanding during the search task. This experiment was performed in accordance with the tenets of the Declaration of Helsinki. IRB #: 14-09-16 - Psychophysical Study of Visual Perception and Eye Movement Control.

Subjects underwent a vision screening procedure before the experiment (for details, see Bex & Skerswetat, 2021). They were tested for acuity, color detection, and depth. These tasks were presented in random order for each subject. All subjects had normal acuity and color vision; four subjects had deficiencies in at least one spatial frequency during our depth task.

### Procedure

#### Experiment 1: Simulated task

In our first study, subjects (N = 40) were given a simulated task to carry out when viewing a scene. There were ten scenes, five indoor and five outdoor, taken from the LabelMe database (Russell et al., 2008). Each scene was assigned two possible tasks, for example, in a kitchen scene, the tasks were either “make a cup of tea” or “unload the dishwasher.” Each subject randomly received one of these two tasks, and tasks were balanced across subjects. Subjects were instructed to view the scene as if they were accomplishing the task. Subjects were instructed to press the spacebar when finished with each imaginary task. Analysis for the simulated task focused only on semantic salience. We calculated a semantic salience heatmap for the two simulated tasks for each image; each heatmap represented the similarity between each object and each task. To generate scene-context labels, we used the task as our scene descriptor. Because GloVe does not accept multiple words as a single input, we broke our tasks down into only the main words excluding prepositions (e.g., “Read a book by the fire” became “Read,” “book,” and “fire”), then calculated the individual heatmaps for each word and averaged those maps together.

**Fig. 1:**
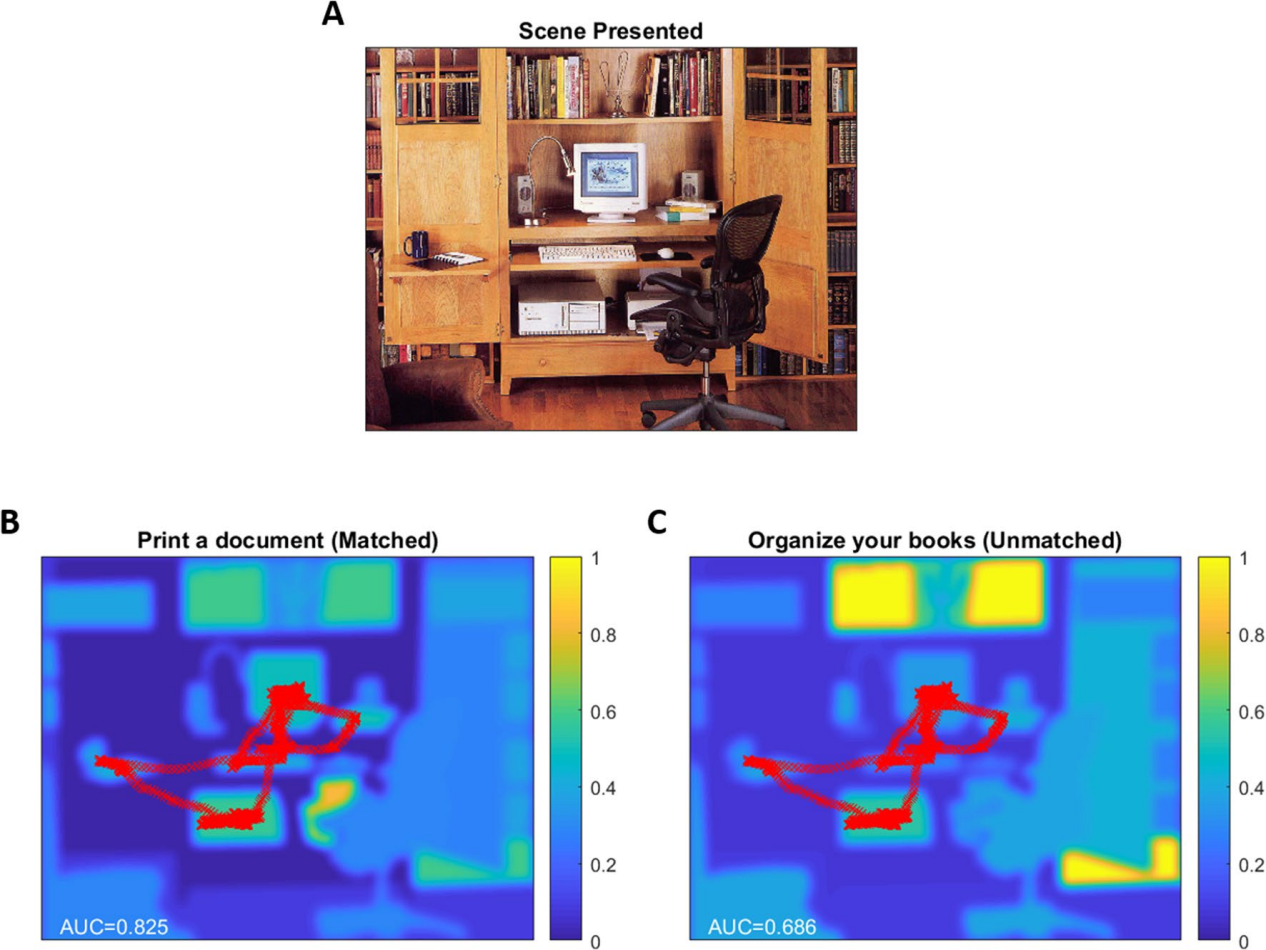
Example of a scene presented to a subject (**A**), the semantic salience heatmap for the task presented to the subject (Matched) (**B**), and the semantic salience heatmap for the task not presented to the subject (unmatched) (**C**). Red X’s represent subject’s gaze (note that in unmatched case (**C**), gaze is reproduced from matched case (**B**), as subject did not perform the unmatched task)

In one analysis, we examined the semantic salience values for image content at the locations of individual fixations during a trial. We used Eyelink’s built in fixation detection to parse out the fixations within each trial. In a further analysis, we used all gaze data to perform an ROC analysis. We calculated the area under the receiver operating characteristic curve (AUROC) score for gaze that overlapped with each semantic salience heatmap. The average fixation and AUROC for each subject and simulated task across all ten scenes/tasks were compared between matched and unmatched (task not presented to the subject) heatmaps (Fig. 1).

#### Experiment 2: Search task

In our second study, subjects (N = 40) viewed 100 scenes (50 indoor, 50 outdoor), while searching for a target object. The name of the target object was displayed as a word in the center of the screen before the scene for 2 s and was then replaced with the search scene, thus all subjects began the task with fixation at the screen center. Subjects were instructed to search the scene for the target object and use the mouse to click on the object once located. Target objects were present in 50% of trials. If the target object was not present, subjects were instructed to click a box labeled “no object” that was below the scene. In the target-absent trials, the target was either high relevance (25%) or low relevance (25%). Feedback was provided between trials. There was a break after 50 trials, and the eye tracker was recalibrated after the break. Recalibration could occur at any point in between trials, if the experimenter supervising the Eyelink monitor noticed significant drift or loss of tracking.

To generate scene context labels, we used PlacesCNN (Zhou et al., 2014) to generate the top five most likely scene labels for each image. PlacesCNN is a convolutional neural network that contains over 7 million labeled scenes and is capable of generating scene labels for newly provided images. The mean semantic similarity of the top five scene labels was used to estimate the similarity of each object to the scene in which it was located.

We took care to ensure the target-present and target-absent conditions were equally weighted with semantic relevance. For each scene, we listed all other scenes that had at least one matching scene label from the top five matches provided by PlacesCNN. We then found the object similarity values for all objects in the target scene, as well as all object similarity values in each non-target scene with a matching scene label. To select a target-absent search object, we cross-referenced these objects and their similarity scores to each scene and chose the target-absent object with the highest similarity with a score within 5% similarity within the target scene (but was not present in the scene). For example, if a target scene had the label “kitchen,” we searched all other scenes with a “kitchen” label for potential target-present objects and target-absent objects. Target-absent objects were required to have a similarity score with the scene label that was within 5% of the target-present object in the target scene but was not present in the target scene. These were considered “high-relevance” targets. Based on this criterion, we ensured that the target-present and target-absent objects were equally semantically relevant in the scenes presented.

We also chose an additional set of target-absent objects that were within 15% semantic relatedness to the present object. These were considered “low-relevance” targets. Because the potential target objects were taken from other scenes with similar object labels, these targets were similar enough within the scene that they were not highly improbable objects (e.g., a penguin in a kitchen), but they were less relevant than the object-present and high-relevant object-absent targets. For example, in one kitchen scene, the present object was “stove” (similarity = 0.450), the high-relevance absent object was “dishes” (similarity = 0.454), and the low-relevance absent object was “bucket” (similarity = 0.308).

We manually checked the most relevant objects to ensure they were suitable targets. We started with the most relevant (highest semantic similarity to the scene label) target-present object and worked down. If there was an issue with the most relevant object (e.g., corresponding target-absent object was present in the scene but unlabeled, target was ambiguous or could be mistaken for a different object, there were multiple versions of the object within the scene, etc.), we checked the next most relevant object, and continued until a suitable pair of objects was found.

**Fig. 2:**
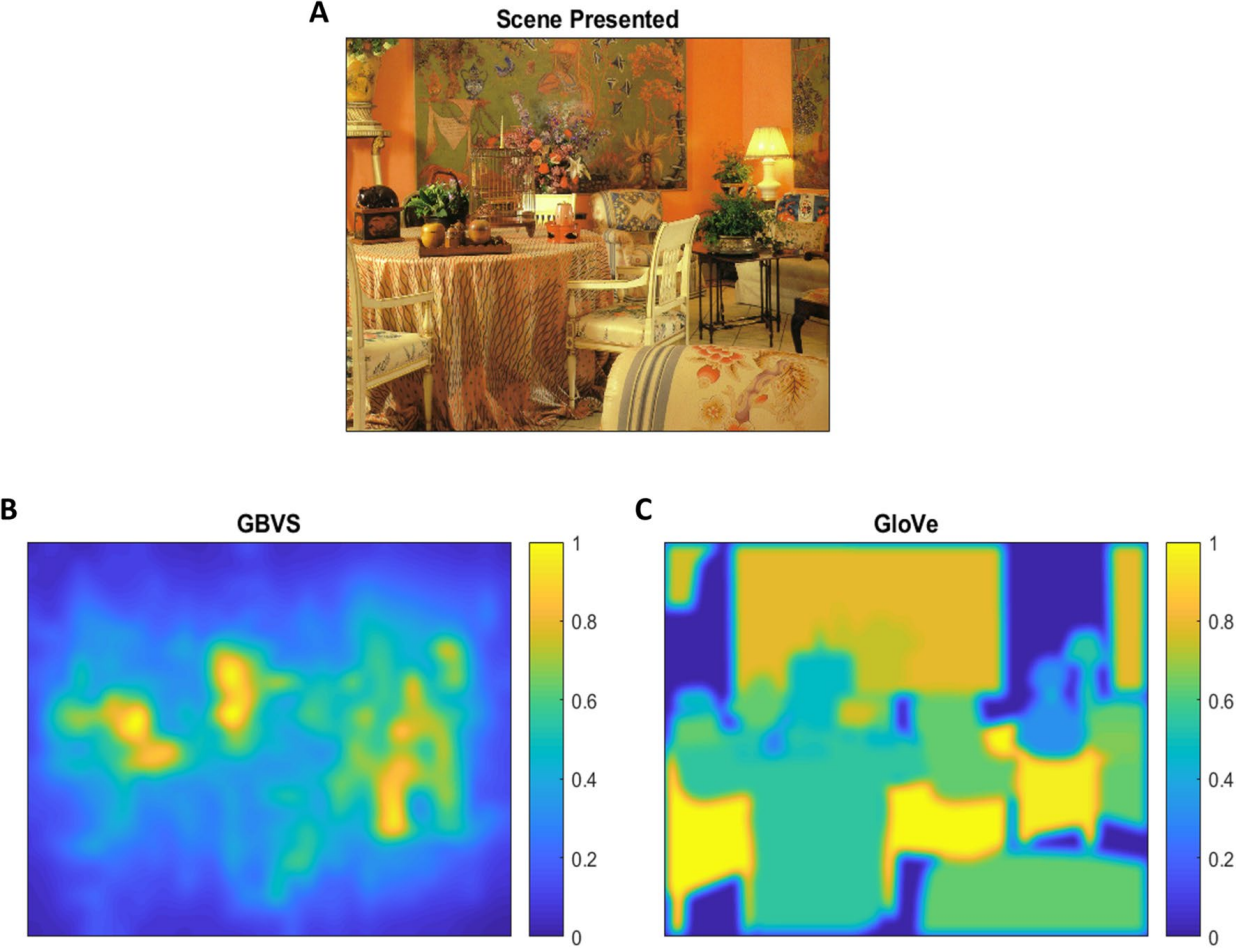
Example of a presented scene (**A**) and its corresponding image salience (GBVS; **B**) and semantic salience (GloVe; **C**) heatmaps

As in the simulated task, we calculated both fixation and AUROC scores for both image salience and semantic salience heatmaps. On average, the GBVS model produces heatmaps with more low-salience areas compared to the GloVe model. In order to normalize the image salience and semantic salience maps (to allow better comparison between methods), we divided our image salience maps into the same number of discrete areas as the number of objects in the matching semantic salience map. This was performed to ensure an equal distribution of discrete areas between both map types. This was done by generating a linearly spaced vector with a number of points equal to the number of objects in a given scene, then grouping the image salience heatmap into the levels specified by that vector. We applied a Gaussian smoothing (σ = 0.375°) to both maps to accommodate uncertainty in fixation location from eye tracker imprecision (Fig. 2).

## Results

### Simulated task

Out of 400 trials, one trial was removed due to missing eye-tracker data (0.25%).

**Fig. 3:**
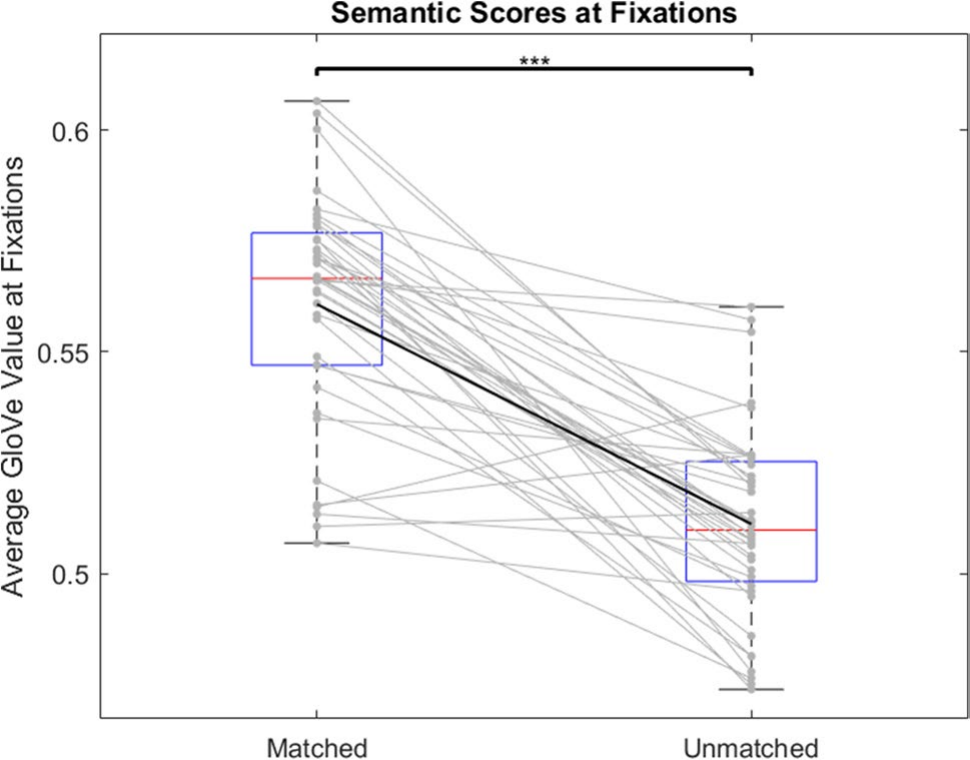
Average salience scores at fixation points for matched and unmatched cases. Gray lines represent difference in average scores for individual subjects. Black line represents mean decrease. Red lines represent median values

A paired-samples t-test found that fixation scores for matched cases were significantly higher than that of unmatched cases (t(39) = 9.714; p < 0.001), and a large effect was found (d = 1.536), suggesting that subjects search a scene in correlation with the semantic content of the task being performed (Fig. 3).

**Fig. 4:**
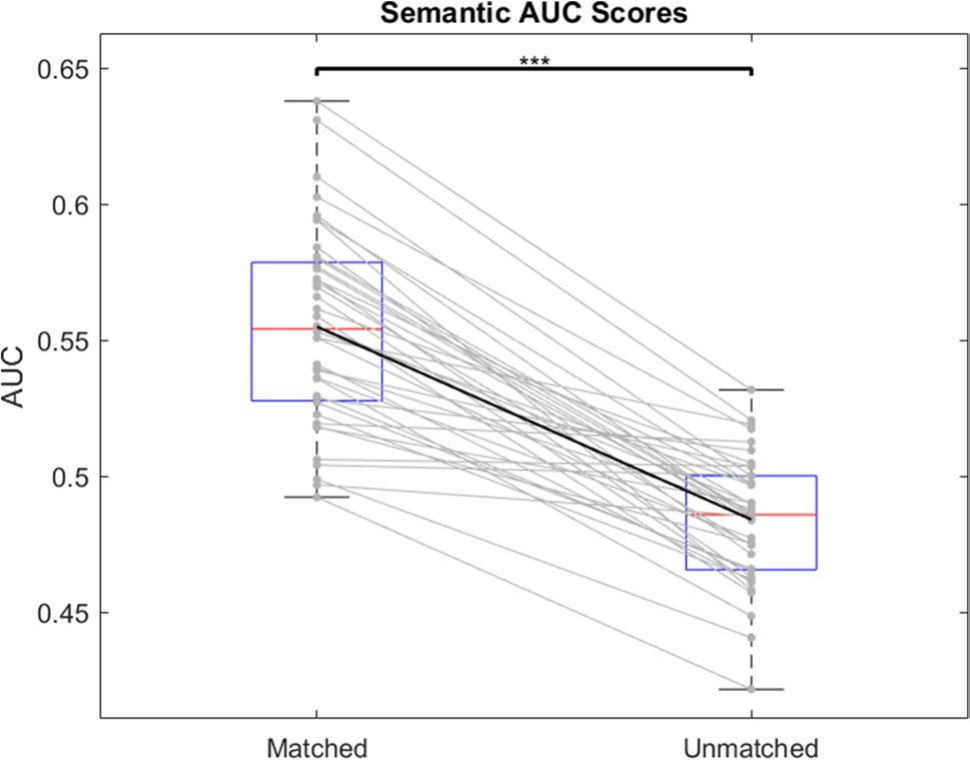
As Fig. 3, for AUROC (area under the receiver operating characteristic curve) scores

Similarly, a paired-samples t-test found that AUROC scores for matched cases were significantly higher than that of unmatched cases (t(39) = 13.083; p < 0.001), and a very large effect was found (d = 2.069), in line with the fixation analysis (Fig. 4).

### Search task

Out of 4,000 trials, 20 trials were removed due to missing eye-tracker data (0.50%).

### Reaction time

A one-way ANOVA showed that reaction time was significantly faster for the target present condition (x̄ = 1.983 s, σ = 1.477 s) than the target absent high semantic salience (x̄ = 4.373 s, σ = 2.581 s) and low semantic salience (x̄ = 4.434 s, σ = 2.716 s) conditions (both p < .001); and the high and low conditions were not significantly different from one another (p = .976).

### Fixations for high and low semantic relevance targets

**Fig. 5:**
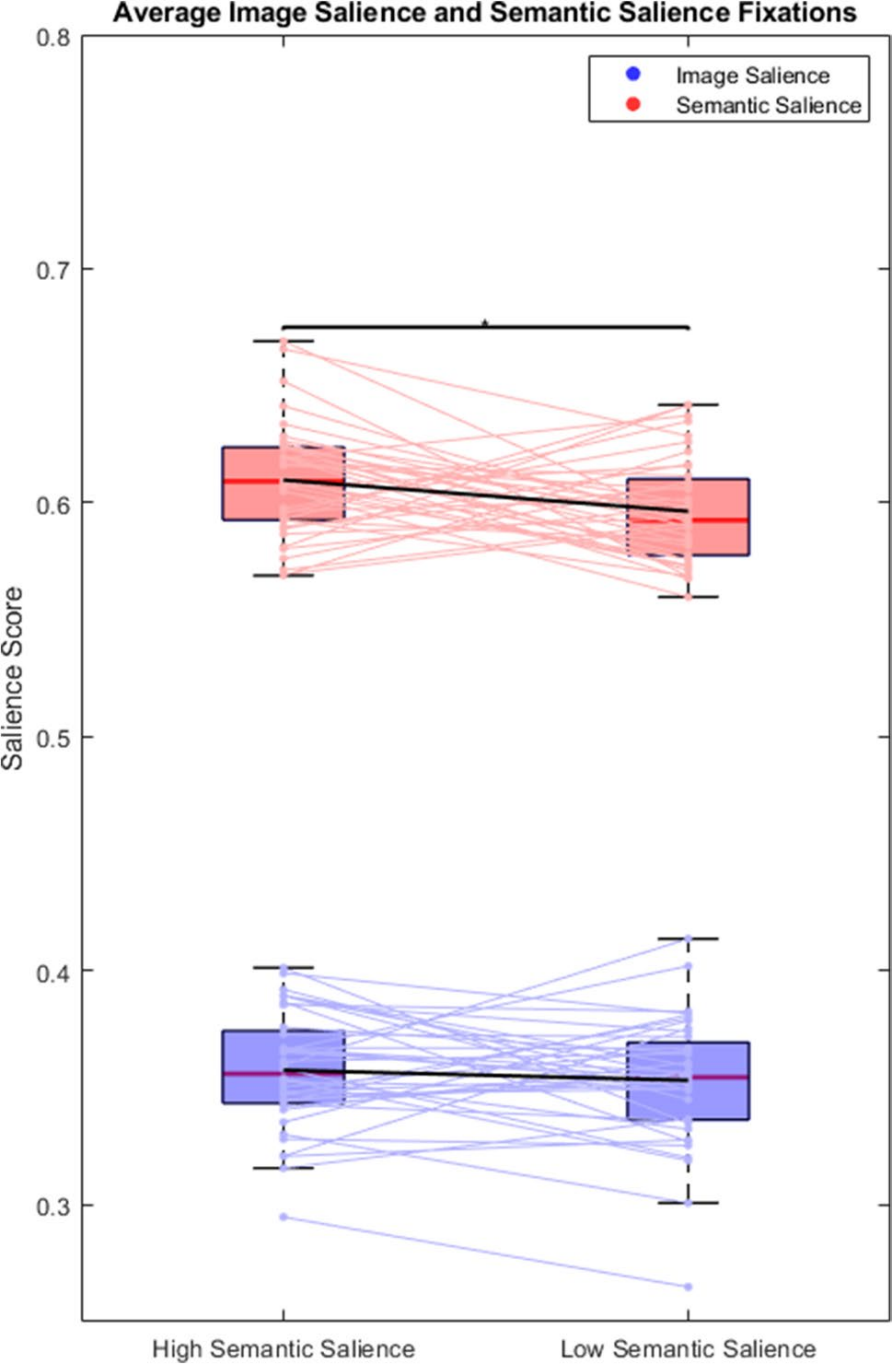
Average salience scores at fixation points for image salience (**blue**) and semantic salience (**red**), separated across target-absent objects with high semantic salience and target-absent objects with low semantic salience. Light blue and red lines represent trends within individual subjects. Red lines within boxplots represent median values. Black lines connecting box plots represent mean values

Figure 5 shows the average image and semantic salience score across all fixations, split across trials where targets had high or low semantic salience. A paired-samples t-test found that semantic salience was an overall better predictor of gaze than image salience (t(39) = -57.089, p < .001), and a huge effect was found (d = 6.383). A paired-samples t-test found that model predictions did not change between target objects with high semantic salience and those with low semantic salience for image salience (t(39) = 0.957; p = .345; d = 0.151), but significantly decreased for semantic salience (t(39) = 2.337; p = .025; d = 0. 370) heatmaps.

For number of fixations, a one-way ANOVA showed that there were significantly fewer fixations made in the target-present condition (x̄ = 6.136, σ = 4.784) than the target-absent high-semantic salience (x̄ = 14.639, σ = 8.710) and low-semantic salience (x̄ = 15.044, σ = 9.118) conditions (both p < .001); and the high- and low-semantic salience conditions were not significantly different from one another (p = .902).

### ROC for high and low semantic relevance targets

**Fig. 6:**
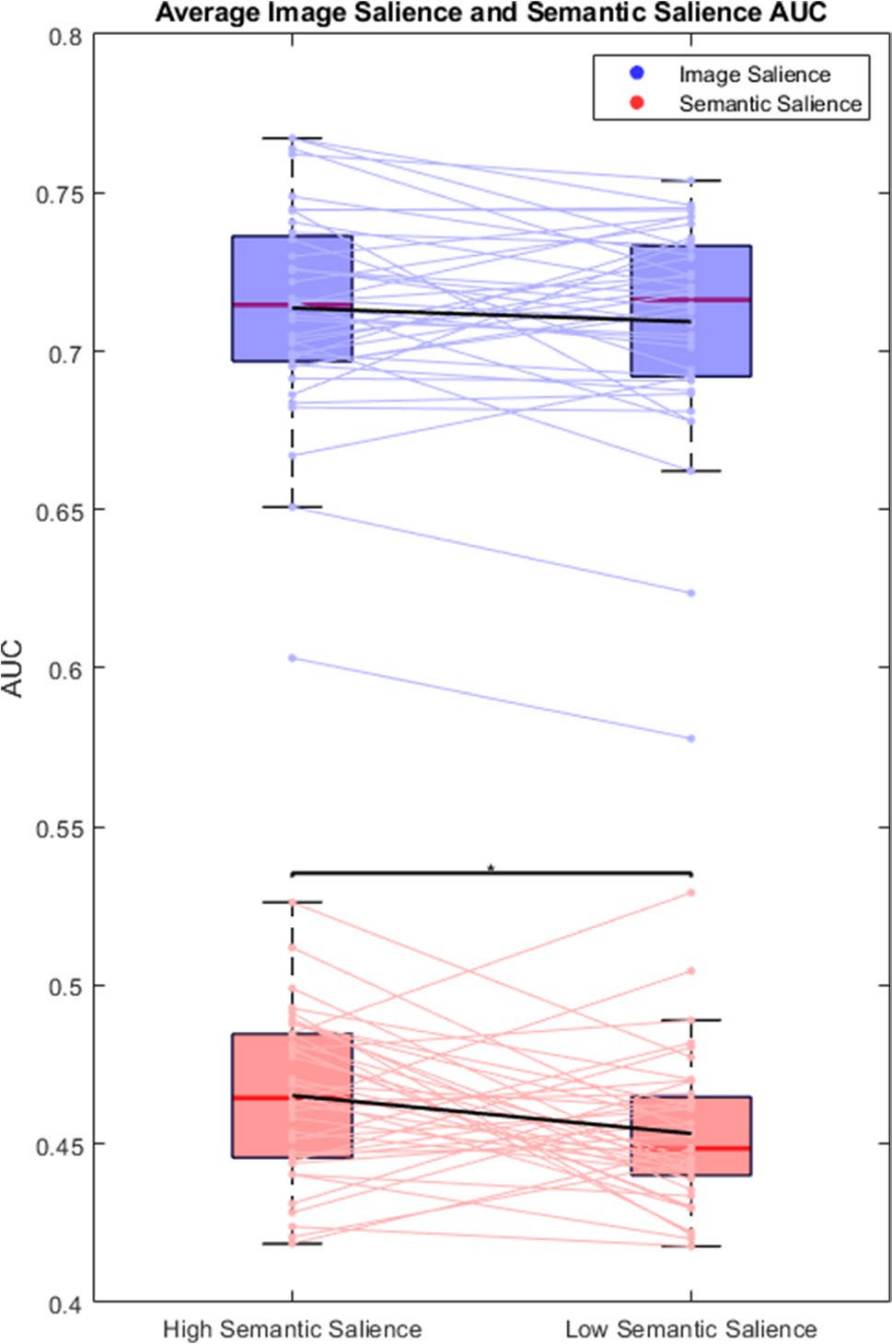
As Fig. 5, for AUC (area under the curve) scores

Figure 6 shows AUC for analysis of fixations predicted by image salience (blue) and semantic salience (red) heatmaps for search targets with high or low semantic similarity with the scene. A paired-samples t-test found that image salience was an overall better predictor of gaze than semantic salience (t(39) = 49.591, p < .001, d = 7.841). A paired-samples t-test found that model predictions did not change between target objects with high semantic salience and those with low semantic salience for image salience (t(39) = 1.130; p = .265; d = 0.179) but significantly decreased for semantic salience (t(39) = 2.304; p = .027; d = 0. 364) heatmaps.

### Fixations across time

**Fig. 7:**
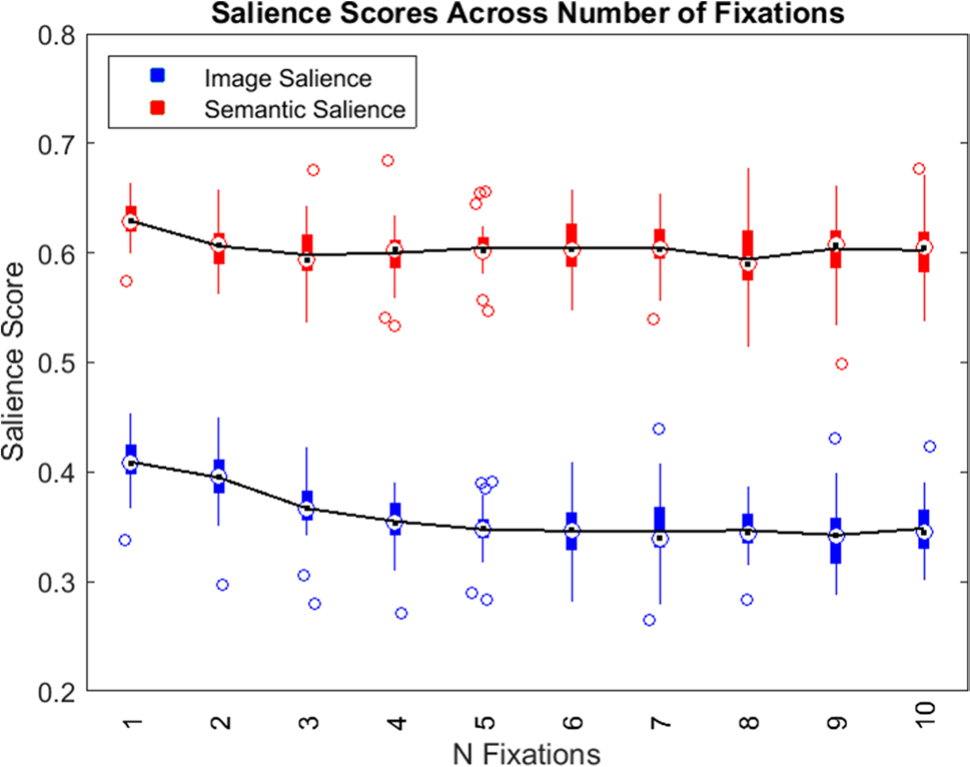
Salience scores for image salience (**blue**) and semantic salience (**red**) across number of fixations. For figure simplicity, only fixation numbers 1 through 10 are displayed. Boxplots represent summary statistics of all subjects at each fixation number. White circles with black centers represent median values. Black lines connecting boxplots represent mean values. Unfilled circles represent outliers

We recorded the salience value at each fixation location for both image and semantic salience heatmaps. For each fixation throughout each trial, we calculated the average salience score across subjects and images. Figure 7 shows boxplots of image salience (blue) and semantic salience (red) for the first to the tenth fixation through the trial. A paired-samples t-test found that semantic salience was an overall better predictor of gaze than image salience (t(39) = -14.631, p < .001, d = 2.313). A linear mixed model analysis showed a significant interaction between salience type and fixation number where image salience decreased with increasing fixation number and semantic salience remained constant throughout trials (X^2^(1, N = 40) = 75.148, p < .001). This analysis also showed a significant interaction between salience type and target condition, where semantic salience increased with target relevance (X^2^(1, N = 40) = 17.029, p < .001).

## Discussion

When viewing scenes, we make multiple fixations that bring the high-resolution fovea onto different locations in the scene for detailed visual analysis. A number of low-level and high-level approaches have been developed to predict the probability that a given location in a scene will be fixated. In Experiment 1 we asked subjects to view scenes as if they were carrying out one of two imaginary tasks. We calculated matched and unmatched fixation and AUROC scores by overlaying subject’s gaze atop the task-relevant heatmap of their performed task (matched) and compared that same gaze to the task-relevant heatmap of the task they did not receive (unmatched). We found that both fixation and AUROCs were significantly and consistently higher for matched cases compared to unmatched cases, confirming many previous reports that task is related to gaze – but here we show that task-related gaze can be quantified using an automated semantic based language analysis without human scoring of semantic similarity. Thus, our results confirm that task can moderate fixation behavior in a manner that cannot be predicted by image salience approaches. It should be noted that while this experiment had far fewer trials than Experiment 2, we still found a robust effect that demonstrates the strong influence of task during gaze. Our results show that language-based models of the semantic relationships among objects and tasks may provide a quantitative mechanism that captures part of this change.

In our second study, subjects completed a present/absent visual search task where targets were of either high or low semantic relevance. In line with many previous studies, participants had significantly higher reaction times and made significantly more fixations when searching for absent targets compared to present targets (for review, see Wolfe et al., 2010). Here we show that subjects searched for approximately the same amount of time and made approximately the same number of fixations when searching for absent targets of both high and low semantic relevance. This suggests that participants were not confounded by low-relevance targets and continued to search for them as long as for high-relevance targets.

Using both image salience and semantic salience heatmaps, we calculated the salience of successive fixations and additionally calculated AUROC scores using all gaze per trial. We found that image salience does not predict gaze differently when searching for high- or low-relevance targets, which is to be expected since there is no a priori reason for them to be lower in feature contrast. However semantic salience is lower for low-relevance targets and therefore is a worse predictor. This makes sense, as high-relevance targets have more predictable locations in the scene based on their normal semantic context (e.g., a dishwasher is typically near a sink in a kitchen, see Võ, 2021), but low-relevance targets do not have the same predictability (e.g., where would a bucket be found in a kitchen? On the counter? On the floor? On a chair?). In another sense, low-relevance targets do not hold strong associations to “anchor objects”. Anchor objects are objects which generally influence the predicted spatial position of other objects (Boettcher et al., 2018; Draschkow & Võ, 2017). For example, a desk may serve as an anchor for a computer, a bowl as an anchor for a spoon, or a sink as an anchor for soap. Low relevance targets in this study are less likely to have anchor objects and thus do not benefit from the semantic priming of anchors, so gaze is not usefully guided by related objects in these scenes. It has been reported that incongruent objects draw gaze when viewing scenes (Coco et al., 2020; Friedman, 1979; Henderson et al., 1999; Öhlschläger & Võ, 2017; Pedziwiatr et al., 2021; Underwood & Foulsham, 2006), however our results demonstrate an additional conclusion: even in the absence of an object, gaze strategies are impacted when searching for a semantically unrelated target.

Additionally, we hypothesized that objects with high semantic similarity with a scene might benefit more from this semantic-based knowledge, while objects with low semantic relevance would benefit less and depend more on an image-based search strategy. However, while we determined that search strategies for objects with high semantic salience are better predicted by semantic salience models than objects with low semantic salience, we found no difference in image salience predictions. Our original hypothesis had assumed that the decline of one strategy would result in the increase of another, however we found that this is not necessarily the case. Upon further reflection, this result is somewhat unsurprising, given that semantic relevance should not affect spatial feature contrast.

We also found that over time, semantic salience remained a consistent predictor of fixations, while image salience became a less reliable predictor after 3-4 fixations. The latter result is consistent with the predictions of image salience approaches, which have demonstrated that image salience is a more prominent factor in search when the image is immediately presented, but as context of the scene begins to become apparent, it becomes less reliable (Parkhurst et al., 2002). In other words, as soon as a scene is presented, image salience is a driving factor in gaze-guidance, but rapidly becomes less utilized once the semantic context is understood (within approximately 3-4 fixations). However, it should be noted that many salience models, including GBVS, exhibit a central bias prediction which can potentially be attributed to this decline. It is possible that the decrease in prediction power of the image salience model is simply an artifact of a tendency to look at the center of the scene at presentation, and then shift fixations around the image as time goes on (Tatler et al., 2005). In the resent study, the word cue for the search target was presented at the center of the display for 2 seconds before each trial. Although observers may have fixated away from the word during the cue phase, participant fixations were therefore biased towards the center of the display, which would potentially increase the AUC for GBVS. Center bias is not a contributing factor in the GloVe model, as it is unrelated to the semantic salience of the objects (Rose & Bex, 2020).

The AUC method has been criticized with the introduction of newer models, many of which are complex neural networks. However, AUC is included in the “similarity cluster” of a variety of different gaze measuring metrics, including Normalized Scanpath Saliency, Pearson’s Correlation Coefficient, Earth Mover’s Distance, and Similarity or Histogram (Bylinskii et al., 2019). In other words, it performs similarly in a pairwise comparison (between 0.80 and 0.99) to these additional metrics. With this in mind, we find it important to discuss the results of our ROC analysis in more detail.

We find that in the ROC analysis, image salience consistently outperforms semantic salience approaches because ROC analyses penalize gaze that does not land in predicted locations. AUC metrics ignore low-valued false positives (Judd et al., 2012), and as a result, using a model that rates the majority of areas as low salience increases the odds of performance. Because all objects in a kitchen are generally semantically kitchen-related, the majority of areas around the scene have high similarity scores with the scene description. Not attending these locations lowers the overall AUC when performing an ROC analysis. In contrast, the GBVS model only rates a few discrete areas as having high image salience, so the model scores much higher. These factors should be considered when performing comparison analyses with these methods, and so an additional comparison is detailed in our fixation-based analyses. The fixation analyses take the raw heatmap value of where all fixations land on a scene and average them together for a mean prediction score. This does not consider false positives or misses, and rather focuses on just what the participant actually viewed during the scene. The limitation of this approach, however, is again the counterbalance of heatmap values between image and semantic salience (where image salience has mainly low values, and semantic salience has mainly high values).

We find this discrepancy between the AUC and fixation analyses important to discuss, as it demonstrates how two methods can produce seemingly opposite results. We also find it important to note that the significant decrease in prediction power of the semantic salience model when searching for low-relevance targets is consistent in both the fixation and AUC analyses. It is interesting that the prediction power of the two models is flipped between these analyses (image salience being better under AUC, semantic salience being better under fixation), but the overall trends between high and low targets remain consistent. Because of this, we stress the trends within the analyses, rather than focus on which model is a better predictor in which context.

Overall, we establish the principal that semantic information carries significance when viewing scenes and that gaze can be guided under the semantic properties of a given task, and our analysis demonstrates that this guidance can be quantified through the application of language models to semantic analysis. We also illustrate that even in the absence of a target object, gaze strategies are still modulated by the semantic relevance of the target search object.

## Data Availability

All data can be found via the Open Science Framework at https://osf.io/ARWNP/.

## Code availability

Data were analyzed using Matlab R2019a version 9.6.0 (The MathWorks, Inc., 2019) and R version 4.1.2 (R Core Team, 2021).
